# Microarray enriched gene rank

**DOI:** 10.1186/s13040-014-0033-1

**Published:** 2015-01-17

**Authors:** Eugene Demidenko

**Affiliations:** Department of Biomedical Data Science, Institute for Quantitative Biomedical Sciences, Geisel School of Medicine at Dartmouth, Hanover, 03755 NH USA

## Abstract

**Background:**

We develop a new concept that reflects how genes are connected based on microarray data using the coefficient of determination (the squared Pearson correlation coefficient). Our gene rank combines a priori knowledge about gene connectivity, say, from the Gene Ontology (GO) database, and the microarray expression data at hand, called the microarray enriched gene rank, or simply gene rank (GR). GR, similarly to Google PageRank, is defined in a recursive fashion and is computed as the left maximum eigenvector of a stochastic matrix derived from microarray expression data. An efficient algorithm is devised that allows computation of GR for 50 thousand genes with 500 samples within minutes on a personal computer using the public domain statistical package R.

**Results:**

Computation of GR is illustrated with several microarray data sets. In particular, we apply GR (1) to answer whether bad genes are more connected than good genes in relation with cancer patient survival, (2) to associate gene connectivity with cluster/subtypes in ovarian cancer tumors, and to determine whether gene connectivity changes (3) from organ to organ within the same organism and (4) between organisms.

**Conclusions:**

We have shown by examples that findings based on GR confirm biological expectations. GR may be used for hypothesis generation on gene pathways. It may be used for a homogeneous sample or for comparison of gene connectivity among cases and controls, or in longitudinal setting.

## Introduction

The key element of Google’s success is the PageRank that determines the order of webpages displayed for a keyword search. The original algorithm by Page *et al.* [1] received considerable attention. The idea to use the PageRank to rank genes with respect to their connection to other genes seems obvious and indeed is not new: GeneRank developed by Morrison *et al.* [2] is a straightforward generalization of PageRank of Google. Two versions of GeneRank are suggested: (1) using GO annotation or (2) Pearson correlation coefficient, *r*. In both cases, the entries of the connectivity matrix are binary: 0 genes are not connected and 1 if genes are connected. In the correlation version (2), the genes defined connected if *r*>0.5. Both versions suffer from series limitations: (1) The GO annotation version is difficult to realize in practice because, e.g. with *n*=50,000 number of genes one has to fill in the connectivity matrix with 2.5×10^9^ elements. Moreover, since the set of genes varies from study to study and from organ to organ, the researcher faces a daunting problem with every new set of microarray experiments. (2) The correlation version brushes off negative correlations which may be very important, and the threshold 0.5 has no justification. The gene rank we suggest is free of these shortcomings: (1) it combines GO library with correlation so that only the connectivity of a small proportion of genes can specified, (2) no thresholding is applied since we use the squared correlation coefficient (coefficient of determination).

The goal of this work is to equip the researcher with a new concept, microarray enriched gene rank (GR), that combines *a priori* knowledge about gene connectivity with researcher-derived microarray data, and can be computed on his/her own personal computer with as many as 50,000 genes and 500 samples within few minutes.

Traditional gene ranking methods using microarray data are based on ordering of *t*-statistics (or respective *p*-values) when the means between cases and controls are compared, or in a more general case, when the microarray sample is correlated with a phenotype, see Winter *et al.* [3], Zuber *et al.* [4]. Much of the literature studies issues related to minimization of the false discovery rate or correlation between genes; see Opgen-Rhein *et al.* [5], Nitsch *et al.* [6], Masoudi-Nejad *et al.* [7]. We, however, consider the gene connectivity problem *regardless* of the phenotype. The assumption of the present work is that gene connectivity can be adequately expressed in terms of the gene pairwise squared correlation matrix, therefore the phenotype is not required. However, the association with phenotype or disease status can be examined further, such as through comparison of GRs between controls and cases.

GR reflects the complexity of genetic organization and is illustrated with several existing microarray data sets. This new genetic quantity gives rise to new biological insights, such as connectivity within clusters/subtypes, between organs within the same organism, and between organisms. GR can be used to discover gene pathways and track those under different experimental conditions or time wise.

## Methods

We assume that the gene expression data are presented as an *n*×*m* matrix, where rows are genes (the number of rows equal *n*) and columns are samples (the number of samples/conditions equals *m*). Hereafter, we use boldface to denote vectors and matrices, and the subscript *i* to indicate the *i*th gene and *j* to indicate the *j*th sample. It is assumed that, for each gene *i*, the sample {*x*_*ij*_,*j*=1,2,…,*m*} consists of independent identically distributed (iid) observations (microarray measurements); moreover, *n* samples belong to a multivariate normal distribution. In practice, we may apply a nonlinear transformation, such as the log-transformation, to avoid skewness, when it is appropriate—say, when observations are positive. Under these assumptions, the *n*×*n* pairwise Pearson correlation matrix computed from the matrix data, **R**={*r*_*ij*_}, is an appropriate measure of genes connectivity; the negative values indicate negative relationships and the positive values indicate positive connectivity. Our concern is the gene connectivity regardless of the negative or positive relationship, so the squared correlation coefficient, or the coefficient of determination, should be used. In fact, the squared correlation coefficient is more interpretable in the statistical sense than the traditional correlation coefficient. Namely, $r_{\textit {ij}}^{2}$ indicates the proportion of the variance of the *i*th gene explained by the *j*th gene and vice verse $\left (r_{\textit {ij}}^{2}=r_{\textit {ji}}^{2}\right)$. If $r_{\textit {ij}}^{2}$ is close to zero, genes *i* and *j* are almost independent; if $r_{\textit {ij}}^{2}$ is close to 1 genes *i* and *j* are almost linearly related.

The premise of this work is that the gene network is represented by an *n*×*n* matrix of squared correlation coefficients, $\mathbf {R}^{2}=\left \{r_{\textit {ij}}^{2}\right \}$. In the terminology of Langfelder and Horvath [8], matrix **R**^2^ is called the co-expression network with *β*=2. Note that **R**^2^ is treated as the whole mathematical object, not the matrix product of **R** and itself. The sum of $r_{\textit {ij}}^{2}$ in the *i*th row of matrix **R**^2^ is an indication of gene connectivity. However, in order to compare genes from different rows, we need to normalize them so that the sum in each row is one. That leads us to the normalized squared correlation coefficient,

(1) $$ r_{\ast ij}^{2}=\frac{r_{ij}^{2}}{\sum_{k=1}^{n}r_{ik}^{2}}.  $$

The *n*×*n* matrix with these entries will be referred to as the *normalized squared correlation matrix*$\mathbf {R}_{\ast }^{2}$. Matrix $\mathbf {R}_{\ast }^{2}$ belongs to the family of *stochastic matrices*: All elements are nonnegative and the sum of elements in each row is one.

Now let *p*_*j*_ represent the rank of gene *j*. Another way to compute the gene connectivity is to use the weighted sum of squared correlations with weights *p*_*i*_: $\sum _{i=1}^{n}p_{i}r_{\ast ij}^{2}$. This means that the squared correlations are weighted with respect to the connectivity and as in the PageRank, leads to a recursive definition of *p*. Let the nonnegative and less or equal to one *a*_*j*_ be the *a priori* gene *j* connectivity measure, 0≤*a*_*j*_≤1. If *a*_*j*_=0, gene expression data adds nothing to *a priori* connectivity; if *a*_*j*_=1, then the connectivity is solely derived from the expression data. Measure *a*_*j*_ may represent our biological knowledge about gene connectivity, or it may be a noninformative *a priori* distribution frequently used in the Bayesian approach, Gelman *et al.* [9].

Finally, the recursive definition of the microarray enriched gene rank is:

(2) $$ p_{j}=\frac{1}{n}(1-a_{j})+a_{j}\sum_{i=1}^{n}p_{i}r_{\ast ij}^{2},\quad j=1,2,\ldots,n.  $$

The first term on the right-hand side, (1−*a*_*j*_)/*n*, represents our *a priori* knowledge about the connectivity of gene *j*. The second term, $ a_{j}\sum p_{i}r_{\ast ij}^{2}$, represents the connectivity derived from the gene expression data at hand. Since *p*_*j*_ combines *a priori* knowledge about gene connectivity with microarray data experiments, we call it the microarray enriched gene rank, or simply gene rank (GR).

In matrix language, Equation (2) can be expressed as **p=H**^′^**p,** where **p** is the *n*×1 vector of gene ranks, ^′^ is the matrix transposition symbol, and **H** is the *n*×*n* matrix

(3) $$ \mathbf{H=}\frac{1}{n}(\mathbf{1-a)1}^{\prime }+\mathbf{A\mathbf{R}_{\ast}^{2}},   $$

where **1** is the *n*×1 vector of ones, **a** is the *n*×1 vector of {*a*_*j*_,*j*=1,..,*n*}, and **A** is the *n*×*n* diagonal matrix with **a** on the diagonal, **A**=diag(**a**).

### **Definition1**.

The gene rank (GR) vector is the maximum left eigenvector of matrix **H**: the *n*×1 vector **p** that satisfies the equation

$$\mathbf{p=H}^{\prime}\mathbf{p.} $$

It is proven in the online methods that matrix **H** is a stochastic matrix, and by the Perron-Frobenius theorem (Berman *et al.* [10]) there exists an eigenvector **p** with nonnegative elements such that **p**^′^=**p**^′^**H**, or equivalently **p=H**^′^**p.** This eigenvector has unit length and corresponds to the unit eigenvalue of matrix **H**^′^**.** Because matrix **H** has all positive elements in our case, this eigenvalue is maximum and other eigenvalues are positive but smaller than one. Hereafter, we refer to GR as the left maximum eigenvector of matrix **H**. In a special case when all gene connectivities are *a priori* set to zero, we have *a*_*j*_=1 and the GR of gene *j* is proportional to the sum of squared correlations in row *j*. In our computations below, we assume the noninformative prior connectivity distribution, *a*_*j*_=0.9=const (Google uses *a*_*j*_=0.85). Our results reported below are fairly robust to the choice of this constant due to large *n*.

When *n* is of the order of a few thousands, standard methods of eigenvector computation may be used. Several authors suggest efficient algorithms for computation of the maximum left eigenvector of a stochastic matrix (Golub and Greif [11], Wu *et al.* [12]). However, when *n* is of the order of tens of thousands, new algorithms are required because even storing the squared correlation matrix is problematic. An efficient method for computation of GR using a public domain statistical package R does not require storing **R**^2^ and is outlined in the online methods with the R [13] script provided. For example, computation of GR for a microarray data with 50,000 genes and 500 samples takes only few minutes on a personal computer.

### Gene rank and cluster analysis

Cluster analysis is a popular method for microarray data to identify groups or subtypes of genes. The most popular cluster techniques are *k*-means and hierarchical clustering, which is usually visualized with a dendogram. In both methods, the Euclidean distance between samples is used. Typically, data normalization is performed prior to clusterization: the mean is subtracted from each row and divided by the norm so that the norm of each row is 1. In this case, the Euclidean distance between normalized samples **z**_*i*_ and **z**_*j*_ can be expressed via the Pearson correlation coefficient, *r*_*ij*_, as

$$\left\Vert \mathbf{z}_{i}-\mathbf{z}_{j}\right\Vert =\sqrt{2}(1-r_{ij}). $$

This formula hints to a close relationship between cluster analysis and our GR. As follows from this formula, it is plausible to expect that genes from the same cluster have high rank because they are close to each other. Similarly, if the density of GR is a mixture of several components, these components may be associated with gene clusters, so the number of components would be equal to the number of clusters. We illustrate this association with several data examples below.

### Connection of a specific gene to other genes

By the definition of the gene rank, we have $p_{i}=\sum H_{\textit {ji}}p_{j}$, which can be interpreted as the decomposition of the *i*th gene rank into *n* connections to the remaining genes. As follows from this formula, we define the connection of the *i*th gene with the *j*th gene on the percent scale as

$$c_{ij}=\frac{p_{j}}{p_{i}}H_{ji}\times 100\%. $$

All *c*_*ij*_ are nonnegative and the sum of *c*_*ij*_ over *j*=1,2,..,*n* is 100%. We illustrate this decomposition with several examples below.

## Results

In this section, we illustrate the computation of the gene rank as the left maximum eigenvector and show how this measure generates insights in our understanding of genes’ connection across organisms and across organs within the same organism.

### Gene rank for studying the survival of diffuse large-B-cell lymphoma patients

The paper by Rosenwald et al. [14] is a pioneering work in which 7,399 gene-expression profiles derived from biopsies of 240 diffuse larger-B-cell lymphoma patients are used to predict cancer survival after chemotherapy. Following this phenotype-based ranking, genes have been prioritized with respect to the *p*-value of the coefficient at the gene expression variable in the Cox proportional hazard model. In other words, a gene had a high rank if it was a good predictor of the survival. The goal of this section is to understand how this phenotype-based ranking is related to our microarray enriched gene rank reflecting the gene connectivity. Specifically, we want to know whether these two gene characteristics are positively (or negatively) correlated.

**Figure 1:**
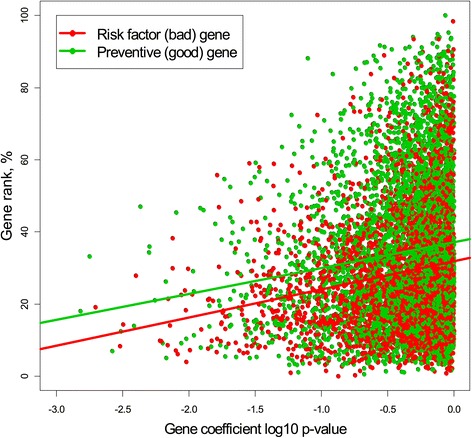
**The relationship between traditional gene ranking, expressed as the** ***p*** **-value of the gene in the Cox proportional hazard model on the log scale, and gene rank.** Risk factor genes are less connected.

The correlation between traditional gene ranking based on the *p* -value and the GR is presented graphically in Figure 1. The *x* -axis corresponds to 7,399 *p*-values from the Cox survival model on the log10 scale and the *y*-axis corresponds to 7,399 GR values on the percent scale, (*p*−*p*_min_)/(*p*_max_−*p*_min_)×100*%*. Different colors are used for risk factors (*bad*) and preventive (*good*) genes corresponding to negative and positive model coefficients, respectively.

The solid lines depict linear regression between two measures for *bad* and *good* genes with approximate equal slope of about 7 but different intercepts (*p*-values <10^−16^). *Good* genes are more connected than *bad* ones, but what is remarkable is that an increase by one order of the *p*-value leads to a 7% increase in the gene connectivity in both groups. One plausible explanation is that *good* genes are more connected since they represent unaffected genes of normal tissue. *Bad* genes, which underwent mutation, do not have enough connection with other genes and therefore their rank is smaller on average. The fact that the regression lines are parallel in both groups has yet to be explained.

**Figure 2:**
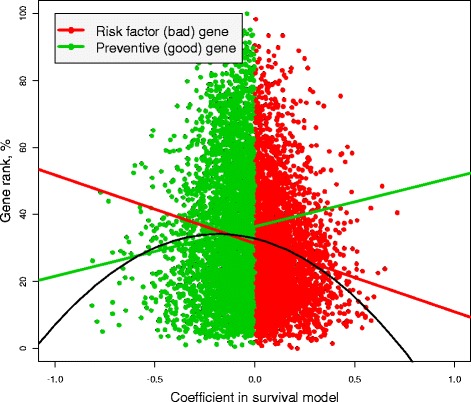
**The relationship between the coefficient in the Cox proportional hazard model (patient survival) and gene rank.** The red and green straight lines are the regressions based on the bad and good genes, respectively; the black line depicts a quadratic regression based on all genes.

Since the *p*-value depends on the ratio of the coefficient to its standard error, it is important to know whether the relationship between the *p* -value and gene connectivity is driven by the coefficient of the Cox model that determines patient survival. To answer this question, we plot GR versus the coefficient, again using different colors to distinguish between bad and good genes; see Figure 2. As in the previous plot, we show two regression lines that reflect the relationship between connectivity and the coefficient in the survival model in two groups. The black line depicts the quadratic relationship between the coefficient and GR (all regression coefficients are highly significant). This analysis confirms our previous finding: the worst and the best genes are less connected.

### Ovarian carcinoma microarray data

Classification of ovarian carcinoma could significantly improve treatment outcomes by identifying the subtype of the ovarian cancer [15]. Considerable effort has been devoted by The Cancer Genome Atlas (TCGA) Research Network researchers to carrying out gene expression experiments to identify clusters of genes that could better classify the disease and predict the treatment outcome [16]. We use the TCGA microarray data set with 11,864 gene microarray expressions obtained from 489 ovary biopsies recently analyzed using cluster algorithm techniques, Verhaak *et al.* [17]. At least four subtypes of genes have been identified with substantial overlap. Below, we suggest gene connectivity analysis based on the GR concept. We hypothesize that genes within the cluster have stronger connectivity and GR density can be used to identify groups of genes.

**Figure 3:**
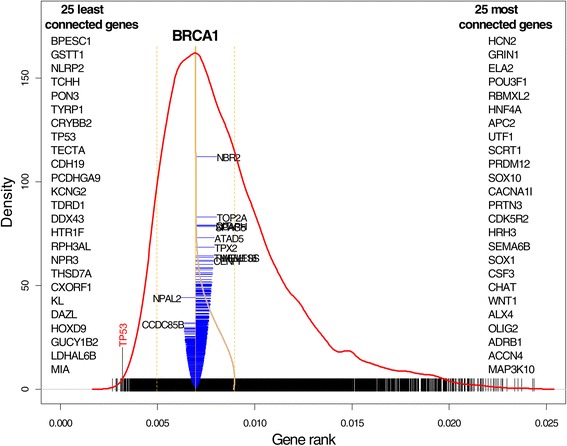
**The GR density distribution for the ovarian TCGA microarray data with 25 the least and 25 most connected genes.** The density distribution for gene BRCA1 is shown in blue with the horizontal bar proportional to the correlation coefficient. The names of 10 genes positively correlated with BRCA1 are shown at right, and the two most negatively correlated genes are shown at left.

We start by computing GR and displaying the density along with the top least 25 and 25 top most connected genes out of 11,864; see Figure 3. These genes are displayed at left and right, respectively. For example, gene BPESC1 is the least connected while gene HCN2 is the most connected. The actual value of GR for each gene is depicted as a black vertical bar: BPESC1 corresponds to the leftmost bar and HCN2 corresponds to the rightmost bar. The red curve depicts the density of the GR distribution with the Gaussian kernel using the bandwidth = 0.00034. This value of the bandwidth, on one hand, reflects deviations in the distribution and, on the other hand, is not too bumpy. The blue cone-like part of the plot depicts the connection of a specific gene, in this particular example gene BCRA1, the most notable gene linked to breast and ovarian cancers, with remaining genes. This gene is the most typical gene in terms of its connectivity and it is mostly connected with gene NBR2. Indeed, this connection is well known from the literature [18], Suen *et al.* [19]. The width of the bar is proportional to the gene’s correlation coefficient with remaining genes. If the correlation is positive the bar extends to the right, otherwise to the left. Dotted lines on both sides correspond to 1 and −1 to facilitate judgment about the strength of the correlation. Also we show the names of 10 positively and two the most negatively correlated genes. As follows from this plot, less correlated genes are at the bottom and more correlated genes are at the top (the yellow curve represents the density of the squared correlation distribution). Two important observations can be drawn from this plot: (1) the GR distribution is not normal and several components can be seen, as either bumps or changes in the density curvature, (2) among 11,863 genes only a handful of genes are highly correlated with BCRA1 (they are located in the top half of the blue plot), and the majority of the those genes are positively correlated.

Several the least connected genes are mentioned in the literature in association with ovarian cancer: The most prominent is TP3 (P53) which mutations lead to various types of cancer as reported in Schuijer and Berns [20]. It was documented by Gates *et al.* [21] that gene GSTT1 produces enzymes which catalyze carcinogens and increase the chance of the ovarian cancer; gene KL is associated with several types of cancer such as breast and lung, besides ovarian cancer; HTR1F is named among genes associated with ovarian cancer, see Cody *et al.* [22].

**Figure 4:**
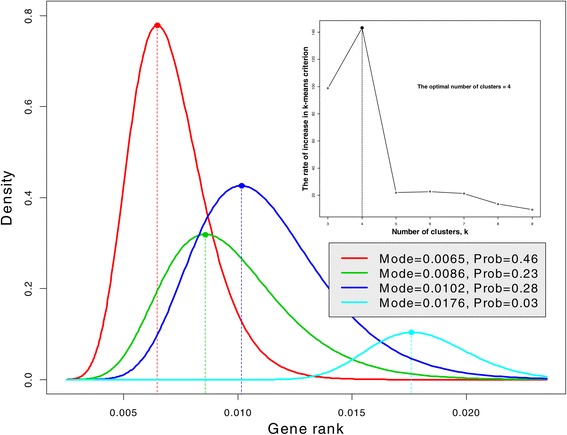
**Gaussian mixture distribution of gene rank for ovarian tumors.** Four density components correspond to four clusters of microarray data found by the *k*-means algorithm. The inset illustrates that the microarray ovarian cancer data form four clusters/subtypes.

Now we draw our attention to the GR distribution. Visual inspection of the GR density (red curve) reveals a possibility of several clusters of gene connectivity; an obvious connectivity cluster is where gene PKD1 is located. As was mentioned above, there are four clusters/subtypes of ovarian cancer genes. To test this finding, we plot the rate of increase of the criterion in the *k*-means algorithm versus *k* to determine at what *k* it reaches its maximum, according to the method of the L-curve; see Hansen *et al.* [23] (inset in Figure 4). According to this analysis ovarian cancer genes form four clusters/subtypes. The question we pose is as follows: Do these clusters impose four clusters in the gene connectivity and how can we determine these clusters using GR? We use the Gaussian mixture distribution technique (Hastie *et al.* [24]) to express the density of the GR distribution, *f*(*p*), using four components:

(4) $$ f(p)=\sum_{i=1}^{k}\pi_{i}\phi \left(p;\mu_{i},{\sigma_{i}^{2}}\right),   $$

where $\phi \left (p;\mu _{i},{\sigma _{i}^{2}}\right)$ denotes the Gaussian density with mean *μ*_*i*_ and variance ${\sigma _{i}^{2}}$ and *π*_*i*_ represents the proportion of genes in the *i*th component, $\sum \pi _{i}=1$. Since the distribution of GR is skewed and positive, the mixture distribution is plotted on the log scale and then transformed back to original values. The result of parameter estimation using the Expectation-Maximization (EM) algorithm (Murphy [25]) is presented graphically in Figure 4. Four clusters/subtypes correspond to four density components depicted with different color. The majority of the genes (the first subtype probability, *π*_1_= 46%, red) have the lowest connectivity with mode = 0.0065; the second and third subtypes (green and blue) have close connectivity with probability close to 25%. Genes from the fourth subtype are closely connected and they constitute the minority (probability, *π*_4_= 3%).

### Gene rank transformation during rice growth

The paper by Fujita et al. [26] examines how microarray gene expressions change during rice reproduction starting from pollination–fertilization and ending with zygote formation. The rice gene expression data comprise *n*=57,381 probes/genes and *m*=99 samples; the data were obtained through the NCBI GEO DataSets website: http://www.ncbi.nlm.nih.gov/gds/. Rice, as well as other plants, goes through four stages of developmental progression: (1) anther development, (2) pollination–fertilization, (3) embryogenesis: the bottom quarter, and (4) embryogenesis: the top three quarters. We use GR to answer the following questions. Does the complexity of the gene network characterized by GR reflect the four transformations? If so, can one quantify the proportion of the gene network built at each stage? The assumption is that the correlation/connectivity between genes changes with the phenotype (in this case stage of the rice development progression). For validation purpose, we act in the reverse fashion: compute correlations using all data and see whether the distribution of GR reflects the four groups (the same approach is used for the *Drosophila* data in the next section). A similar approach has been recently used by Langfelder *et al.* [27].

**Figure 5:**
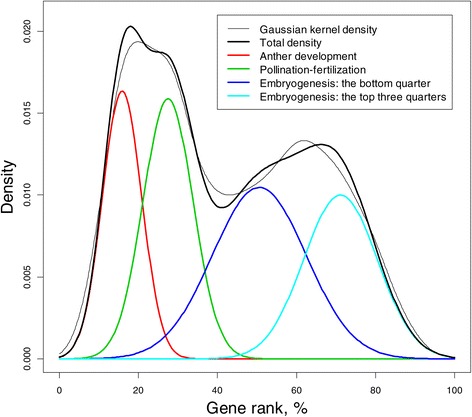
**Identification of four developmental stages of rice using gene rank computed using algorithm described in Theorem 3 of the** Appendix **.** The Gaussian mixture EM algorithm was used to estimate the components of the GR density.

The result of our analysis is presented in Figure 5, where the gene rank, as the left maximum 57,381-dimensional eigenvector of matrix **H,** is computed using the algorithm outlined in Theorem/method 3 of the Appendix. The first two algorithms of GR computation do not work due to the large number of genes; it took eight iterations or about 3 minutes to achieve the accuracy of 10^−5^ on my desktop PC. The GR distribution on the percent scale is represented using the Gaussian kernel density**(**thin line). Clearly, the density is multimodal, indicating that there are several components/clusters in GR distribution. To extract those components, the EM algorithm (Murphy [25]) is used, as in the previous example (4). The results of the estimation are shown in Table 1 and depicted in Figure 5 using a different color for each component. Each component of the mixture corresponds to a specific development phase and is determined by three parameters: *π* = the proportion of the gene network developed at the stage of development, *μ* = the mean connectivity index, and *σ*= standard deviation (SD) of the connectivity. The GR reflects these four rice transformations. Remarkably, the gene rank distribution estimates approximately equal (the proportion is about 25%) what was know from the pre-microarray biology of the plant. The fact that SDs at embryogenesis stage are larger may be explained by the fact that at the last stages of development the gene connections are more complicated compared with the early development and therefore more heterogeneous. However, further investigation is required to fully comprehend the insights from the gene rank results.

**Table 1:** **Gaussian mixture GR distribution of rice growth**

**Developmental stage**	**Mean**	**SD**	**Prop**
	***μ***	***σ***	***π***
Anther development	15.9	4.97	0.203
Pollination–fertilization	27.5	6.32	0.252
Embryogenesis: the bottom quarter	50.6	11.8	0.311
Embryogenesis: the top three quarters	71.1	9.34	0.234

It seems obvious that later rice genome development should increase the complexity of the gene network. Can this statement be supported by our GR distribution at each stage? We use the cumulative distribution function (cdf) to answer this question.

#### **Definition2**.

Let *X* and *Y* be two random variables. We say that *X* is smaller than *Y* in stochastic sense if Pr(*X*≤*z*)≥ Pr(*Y*≤*z*), or in terms of the cumulative distribution function *F*_*X*_(*z*)≥*F*_*Y*_(*z*), for every real *z*.

**Figure 6:**
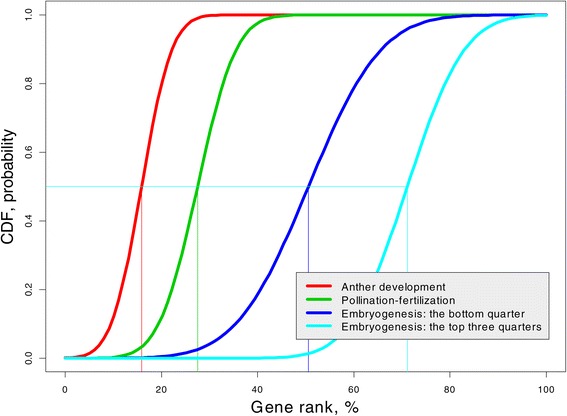
**Cumulative distribution functions (cdfs) for four stages of rice development.** The later development has greater gene connectivity expressed via GR on the entire range.

This definition can be interpreted as follows: For every *z*, the proportion of *X* values smaller than *z* is larger than the proportion of *Y* values smaller than *z*. For example, using this definition, we can state that women are shorter than men in a stochastic sense because the proportion of women is larger than the proportion of men among people of height ≤*z* for each *z*. Note that the inequality between the means does not imply stochastic inequality; however, it can be proven mathematically that stochastic inequality implies inequality between means and medians. The stochastic inequality is the most stringent inequality between random variables. We use this definition to demonstrate that the complexity (connectivity) of genes during rice development increases using the Gaussian cdf GR mixture distribution with parameters shown in Table 1; the results are presented in Figure 6. Indeed, the GR cdf for each consecutive stage shifts to the right indicating that “Embryogenesis: the top three quarters” has the highest and “Anther development” has the lowest gene rank. For example, we may consider the medians of GR for each rice development stage (depicted by thin lines with the appropriate color): While the median GR in the earliest stage is about 15%, the median GR in the latest stage is about 70%.

### Comparison of gene rank across Drosophila organs

**Figure 7:**
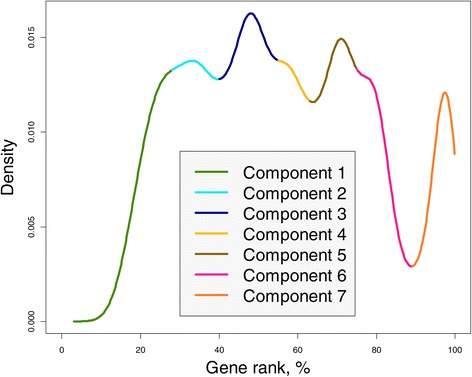
**The seven-component GR density for** ***Drosophila melanogaster*** **.** Each component represents a cluster in the distribution of gene connectivity in eight fly organs.

In this example, we demonstrate that gene connectivity changes from organ to organ in the same organism. We illustrate this phenomenon using the microarray data on *Drosophila melanogaster* (Chintapalli *et al.* [28]), which combines eight organs, brain, head, midgut, tubule, hindgut, ovary, testis, and accessory gland. Our hypothesis is that different organs exhibit different gene connectivities. To test this hypothesis, we compute GR using all samples to see whether its density reflects a multi component character. Indeed, as follows from Figure 7, the distribution of gene rank is multimodal and represents seven clusters of values attributable to different fly organs.

### Comparison of the gene rank across organisms

**Figure 8:**
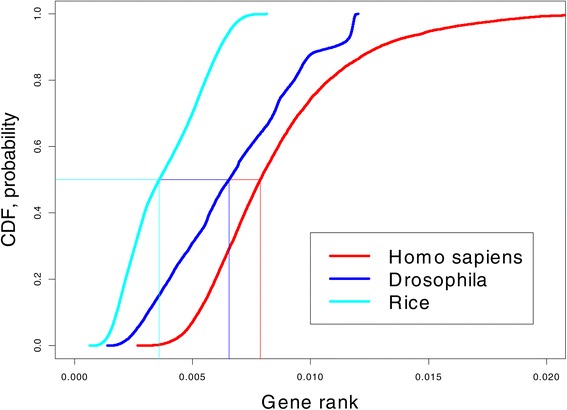
**Comparison of gene connectivity across three organisms using cdf.** The higher the cdf, the more connected are the genes.

The gene network connectivity/complexity should increase from simple to complex organisms. While everybody agrees with this statement, there is no rigorous empirical verification of this conjecture. We tackle this question using microarray data from the three experiments, rice, *Drosophila* and *Homo sapiens*, analyzed previously, by plotting the empirical GR cdfs on the same graph; see Figure 8. Following definition of stochastic inequality, rice has the smallest gene connectivity and *Homo sapiens* has the largest connectivity (the median GR is where the cdf = 0.5). Remarkably, there exists a strong separation between the three organisms despite the fact that gene expressions represent different organs at different stages of development.

## Conclusions

We have introduced a new concept that reflects gene connectivity, the gene rank (GR). GR augments our *a priori* knowledge about gene connection using microarray experiment data. We have shown by examples that the new measure reflects biological expectations and may lead to insights of gene network development in time, across organs of the same organism and across organisms. GR may be used for hypothesis generation in establishment of gene pathways. For example, the fact that many least connected genes are among genes directly associated with ovarian cancer is illuminating.

Traditional gene ranking based on the *t*-test or, in general, based on correlation with a phenotype and our GR look at the microarray data matrix orthogonally. The phenotype approach uses the horizontal correlation of genes with phenotype. In contrast, GR reflects the vertical correlation across genes, so the phenotype information is not used. Both angles of microarray data analysis are important and valid. However, we believe that GR is more fundamental from the biological point of view, although the *t*-test may be more clinically important.

We have shown that GR can be used for identification of gene hubs (Choi and Kendziorski [29], Wu *et al.* [30]) as an alternative to cluster analysis. This cross-gene measure can be used to modify the *t*-test and the respective threshold for the *p*-value to reduce the false discovery rate in genome-wide association studies; see Zuber and Strimmer [4], Yassouridis [31].

In this work, a simple constant *a priori* assumption (constant damping factor) was used for gene connectivity. More work must be done to enrich the GR computation with available gene network connection information such as GO or IMP (Wong *et al.* [32]). For example, if *A*_*ij*_ is a binary connectivity matrix build on the basis of GO annotations (*A*_*ij*_=1 if genes *i* and *j* are connected and 0 otherwise, assuming that *A*_*ii*_=1) we may set $a_{j}=1- \sum _{i=1}^{n}A_{\textit {ij}}/\sum _{i=1}^{n} \sum _{j=1}^{n}A_{\textit {ij}}$. Use of *a priori* biological information may leverage the possibility of false correlation especially important in the case of a small sample size, *m*.

We have presented just a small amount of empirical evidence that GR can be used to characterize the complexity of genes’ connections in organs and organisms. More studies are required to understand its usefulness as a characteristic of genetic complexity.

## Appendix

### Proof that matrix **H** is a stochastic matrix

We have to prove that the entries of **H** are positive and the elements in each row add to one. The fact that elements of matrix **H** are positive follows from the fact that *a*_*j*_ are positive and less than 1. Now we prove that the sum of elements in each row is 1, or in matrix language that **H1=1.** First, we observe that $\mathbf {R}_{\ast }^{2} \mathbf {1 = 1}$ since the sum of elements in each row of matrix $\mathbf {R}_{\ast }^{2}$ is 1. Second, since **1**^′^**1**=*n* and **A1=a,** we have

$$\begin{array}{@{}rcl@{}} \mathbf{H1} &\mathbf{=}&\left(\frac{1}{n}(\mathbf{1-a)1}^{\prime }\mathbf{\ +AR}_{\ast }^{2}\right) \mathbf{1=}\frac{1}{n}(\mathbf{1-a)1}^{\prime } \mathbf{1+AR}_{\ast }^{2}\mathbf{1} \\ &=&\frac{1}{n}(\mathbf{1-a)}n\mathbf{+A1=(1-a)+a=1,} \end{array} $$

matrix **H** is a stochastic matrix and, therefore, the Perron-Frobenius theorem applies.

### Computation of the left maximum eigenvector

Below we describe three methods for computation of GR under three assumptions regarding the size of the microarray matrix. In all computations the noninformative prior on gene connectivity is used with constant *a*=0.9. In case of an informative prior, vector **a** should specify connectivity between 0 and 1.

**1. Using the built-in function eigen in R for small***n*. When the number of genes is fairly small, say, *n*<5000 computation of the left maximum eigenvector can be done using a built-in function in various software packages. For example, in R this function is eigen.



Note that if **p** is an eigenvector −**p** is an eigenvector. too. Therefore, we take the absolute value of the eigenvector of **H**^′^ in the last line.

**2. The power method for moderate***n*, say, 5000<*n*<10000. It is well known that the left maximum eigenvector with the unit eigenvalue can be computed iteratively as

$$\mathbf{p}_{k+1}=\mathbf{H}^{\prime }\mathbf{p}_{k},\quad k=0,1,\ldots $$

This method is referred to as the power method [33]. The following R code realizes this method starting from **p**_0_=**R**^2^**1/**∥**R**^2^**1**∥. As in the previous method we set *a*=0.9;eps and maxit specify the convergence tolerance and the maximum number of iterations.



Typically, this algorithm requires less than 10 iterations to converge. Moreover, for fairly large *n*, say, *n*>1000 it may be even faster and more precise then using the eigen function because eigen computes all eigenvalues and eigenvectors, but the power method computes only the maximum eigenvector.

**3. Computation of the gene rank for large***n*, say, *n*>10000. As in many datasets, even computation of an 50000×50000 correlation matrix using the built-in function cor becomes troublesome (R reports an error due to lack of memory). Below, we suggest an algorithm based on the power method without computation of the correlation matrix. This algorithm is especially effective when *n* is large and *m* is relatively small, say, *m*<500. For example, it took 3 minutes to compute GR for the rice data usint R version 3.0.1 on my *Acer* laptop (Windows 7, 64 bit, 4 GB RAM, processor Intel(R) CPU @ 1.60 GHz).

In order to avoid computation of a large-size **R**^2^, we need to compute the normalized gene expression matrix **X,** by subtracting the mean over samples and dividing by the norm. Specifically, compute

(5) $$ \mathbf{z}_{i}=\frac{\mathbf{x}_{i}-\overline{x}_{i}\mathbf{1}_{m}}{ \left\Vert \mathbf{x}_{i}-\overline{x}_{i}\mathbf{1}_{m}\right\Vert },   $$

so that the rows of the *n*×*m* matrix **Z** are $\mathbf {z} _{i}^{\prime }$. Importantly, computation of the normalized matrix, **Z** may be accomplished in a vectorized fashion in R with no loop and therefore very efficiently, as shown below.



Then, obviously, **R**=**ZZ**^′^. In the theorem below, we express **R**^2^**1** and more generally $(\mathbf {R}_{\ast }^{2})^{\prime }\mathbf {p}$ without computation of **R**^2^**.** The formulas require a double loop over *m*, but for small *m*, it is more efficient than computation of a large **R**^2^.

#### **Theorem1**.

Let the *n*×*n* correlation matrix be expressed as **R**=**ZZ**^′^ and **R**^2^ be the squared correlation matrix. Then the following representation holds

(6) $$ \mathbf{R}^{2}\mathbf{1}=\sum_{k=1}^{m}\sum_{l=1}^{m}g_{kl}\mathbf{v}_{kl},   $$

where

$$g_{kl}=\mathbf{Z}_{\cdot k}^{\prime }\mathbf{Z}_{\cdot l},\quad \mathbf{v} _{kl}=\mathbf{Z}_{\cdot k}\odot \mathbf{Z}_{\cdot l}, $$

**Z**_·*k*_ and **Z**_·*l*_ are the *k* th and the *l* th columns of the normalized matrix, **Z**, and ⊙ represents the element-wise vector or matrix multiplication. Further, if **p** is any *n*×1 vector, the following representation holds.

(7) $$ \left(\mathbf{R}_{\ast }^{2}\right)^{\prime }\mathbf{p=}\sum_{k=1}^{m} \sum_{l=1}^{m}h_{kl}\mathbf{v}_{kl}   $$

where

$$h_{kl}=\mathbf{Z}_{\cdot k}^{\prime }\left(\mathbf{Z}_{\cdot l}\odot \frac{ \mathbf{p}}{\mathbf{R}^{2}\mathbf{1}}\right). $$

#### *Proof*.

Represent

$$\mathbf{R=}\sum_{k=1}^{m}\mathbf{Z}_{\cdot k}\mathbf{Z}_{\cdot k}^{\prime }. $$

This implies

$$\mathbf{R}^{2}=\sum_{k=1}^{m}\sum_{l=1}^{m}\left(\mathbf{Z}_{\cdot k}\mathbf{Z} _{\cdot k}^{\prime }\right)\odot \left(\mathbf{Z}_{\cdot l}\mathbf{Z}_{\cdot l}^{\prime }\right). $$

Now we use the formula (**ab**^′^)⊙(**cd**^′^)=(**a**⊙**c)(b**⊙**d)**^′^, where **a,b,c,d** are vectors [34]. This leads to the representation

$$\mathbf{R}^{2}=\sum_{k=1}^{m}\sum_{l=1}^{m}(\mathbf{Z}_{\cdot k}\odot \mathbf{Z}_{\cdot l})(\mathbf{Z}_{\cdot k}\odot \mathbf{Z}_{\cdot l})^{\prime }. $$

so that

$$\mathbf{R}^{2}\mathbf{1=}\sum_{k=1}^{m}\sum_{l=1}^{m}(\mathbf{Z}_{\cdot k}\odot \mathbf{Z}_{\cdot l})(\mathbf{Z}_{\cdot k}\odot \mathbf{Z}_{\cdot l})^{\prime }\mathbf{1} $$

But $(\mathbf {Z}_{\cdot k}\odot \mathbf {Z}_{\cdot l})^{\prime }\mathbf {1=Z} _{\cdot k}^{\prime }\mathbf {Z}_{\cdot l}=g_{\textit {kl}}$ leading to formula (6). To prove formula (7), we rewrite $(\mathbf {R}_{\ast }^{2})^{\prime }\mathbf {p=R}^{2}\mathbf {p}_{\ast }$, where **p**_∗_=**p/(R**^2^**1)** assuming the element-wise division, and **R**^2^**1** is defined by (6). But

$$\mathbf{R}^{2}\mathbf{p}_{\ast }\mathbf{=}\sum_{k=1}^{m}\sum_{l=1}^{m}(\mathbf{Z}_{\cdot k}\odot \mathbf{Z}_{\cdot l})(\mathbf{Z}_{\cdot k}\odot \mathbf{Z}_{\cdot l})^{\prime }\mathbf{p}_{\ast } $$

finally leading to formula (7). The theorem is proved.

Below, we show the R code that implements this algorithm.


